# Intrinsically Biocompatible Polymer Systems

**DOI:** 10.3390/polym12020272

**Published:** 2020-01-29

**Authors:** Marek Kowalczuk

**Affiliations:** Centre of Polymer and Carbon Materials Polish Academy of Sciences, 34 M. Curie-Sklodowska St., 41-800 Zabrze, Poland; marek.kowalczuk@cmpw-pan.edu.pl; Tel.: +48-322-716-077-(225)

Polymers are everywhere, even inside of the human body. Polymers can be produced by living organisms, in which case they are called biopolymers, while polymers which possess the ability to be in contact with a living system without producing any adverse effect are referred to as polymeric biomaterials [1]. Polymeric biomaterials may be of natural or synthetic origin. 

The term “biocompatibility” reflects the ability of a polymer material to perform with an appropriate host response in a specific application. Thus, biocompatibility is a property of a polymeric biomaterial–host system [2]. The definition of biocompatibility was redefined over ten years ago, and is now accepted to refer to: “the ability of a biomaterial to perform its desired function with respect to a medical therapy, without eliciting any undesirable local or systemic effects in the recipient or beneficiary of that therapy, but generating the most appropriate beneficial cellular or tissue response in that specific situation, and optimising the clinically relevant performance of that therapy” [3]. The mechanism of biocompatibility is not yet well understood; however, countless attempts have been made to elucidate the framework of mechanisms that controls the events that occur when a biomaterial is exposed to tissue in a human body [4].

The range of biological hazards of polymeric biomaterials is wide and complex. The ISO 10993-1 addresses the determination of the effects of medical devices on tissues, mostly in a general way, rather than in terms of specific devices [5]. Therefore, the biosafety of polymeric biomaterials needs to be predictable, in order to undertake assessments of the potential complications arising from their usage and on the formation of their degradation products. Forensic engineering of advanced polymeric materials (FEAPM) deals with the evaluation and understanding of the relationships between their structure, properties, and behavior, before, during, and after their practical application. Both *ex ante* investigations and *ex post* studies are needed in order to define and minimize any potential failure of novel polymeric biomaterials in specific applications. These elements in the FEAPM methodology are currently being studied in the area of polymeric biomaterials [6].

This book comprises 15 chapters, each of which was published previously as original research contributions of the Polymers Special Issue devoted to biocompatible polymer systems: https://www.mdpi.com/journal/polymers/special_issues/biocompatible_polymers?view=compact&listby=date.
